# TFP-Net: A temporal-feature-prototypical network for CRM optimization and cold-start mitigation

**DOI:** 10.1371/journal.pone.0345461

**Published:** 2026-04-02

**Authors:** Yixuan Li, Jing Dong, Ruoke Wang

**Affiliations:** 1 College of Liberal Arts and Social Science, City University of Hongkong, Hongkong, China; 2 Columbia University, New York, New York, United States of America; 3 Department of Mathematics, London School of Economics and Political Science, London, United Kingdom; Beijing University of Technology, CHINA

## Abstract

With the continuous growth of user behavior data on e-commerce platforms, effectively predicting user behavior, providing personalized product recommendations, and addressing the cold-start problem have become key challenges in recommendation systems. To address these issues, this paper proposes a novel customer relationship management model, TFP-Net, which integrates Temporal Graph Attention Mechanism (TGAT), Deep Feature Interaction Module (DeepFM), and Prototypical Network (ProtoNet) to enhance the performance of recommendation systems. Specifically, TFP-Net uses TGAT to capture the temporal features of user behavior, handling the dynamic changes in user actions across different time periods. The DeepFM module learns the complex non-linear relationships between users and products, while the ProtoNet optimizes recommendations in cold-start scenarios, mitigating the data sparsity issues for new users and products. In the experiments, we evaluate the model on the Taobao User Behavior Dataset and the Amazon Product Dataset. The results demonstrate that TFP-Net outperforms traditional baseline models on both datasets, especially in cold-start scenarios, with a performance improvement of 1.5% to 2.1% over the best existing models, namely DGN-JBP on the Taobao dataset and GACE on the Amazon Product Dataset. TFP-Net achieves an accuracy of 87.8% on the Taobao dataset and 88.1% on the Amazon dataset. Additionally, the model demonstrates superior computational efficiency, with lower training time and inference latency compared to other models, proving its potential in large-scale data processing. TFP-Net effectively addresses user behavior prediction and product recommendation on e-commerce platforms, excelling in cold-start scenarios and computational efficiency. It provides a new solution for personalized recommendations and customer relationship management.

## Introduction

Driven by the wave of digital transformation, CRM systems have become a core pillar of modern business operations. Early CRM systems mainly relied on traditional statistical analysis methods, such as logistic regression and collaborative filtering algorithms. Although these methods perform stably in specific scenarios, they are difficult to capture the complex nonlinear relationship in customer behavior [1,2]. With the development of machine learning technology, algorithms such as Support Vector Machine (SVM) and random forest have been introduced into the field of CRM, which improves the prediction accuracy to a certain extent. However, these algorithms still can’t effective treatment sequence data and network [3,4]. The rise of deep learning has opened up new possibilities for CRM systems. In particular, recurrent neural networks (RNN) and Long Short-Term memory networks (LSTM) have shown their advantages in processing sequential data, providing new solutions for modeling dynamic customer behavior [5].

Currently, breakthrough advances in artificial intelligence, particularly in deep learning, provide strong technical support for the intelligent upgrading of CRM systems. Traditional deep learning methods face clear limitations when handling customer relationship networks because they cannot effectively model the complex interactions between entities [6]. This shortcoming has led researchers to focus on Graph Neural Networks (GNNs), which have unique advantages in processing relational data. Early Graph Convolutional Networks (GCNs) achieved basic graph structure learning through neighbor aggregation mechanisms but underperformed when handling heterogeneous and dynamic graphs [7,8]. Later developments such as Graph Attention Networks (GATs) improved model performance by introducing attention mechanisms but still failed to fully consider the temporal dynamics. The latest temporal graph networks can capture time-evolutionary features but still have room for improvement in feature interactions and cold-start issues [9,10]. Existing research still shows significant room for improvement in the following three key dimensions, which directly correspond to the core pain points of practical CRM business scenarios: First, in temporal modeling, most methods struggle to simultaneously capture short-term fluctuations and long-term trends, making it difficult for CRM systems to dynamically track customer preference changes and identify churn risks in real time; second, in feature interaction, existing models have limited expressiveness for higher-order nonlinear relationships, hindering the implementation of precision marketing strategies for sparse customer data (e.g., low-activity users); and finally, in model practicality, the cold-start problem and insufficient interpretability constrain commercial applications, especially in new customer onboarding and new product launch scenarios where CRM systems urgently need to provide rapid and accurate decision support. These technological bottlenecks directly impact the predictive accuracy and business value of CRM systems.

To address the key challenges in current research and align with the core business demands of CRM systems, this study proposes an innovative Temporal-Feature-Prototypical Network (TFP-Net). This model integrates an enhanced Graph Attention Mechanism, a multi-level feature interaction network, and a prototypical learning framework, enabling comprehensive modeling of the entire customer lifecycle behavior. Designed as a task-specific solution for CRM optimization, TFP-Net establishes a direct mapping between its modular architecture and key CRM workflows: the temporal-aware component effectively captures the dynamic evolution of customer behavior, supporting dynamic customer segmentation and churn prediction in CRM; the feature-crossing network delves into the complex relationships between static attributes and dynamic behavior, laying the foundation for precision marketing and personalized recommendation in CRM; the prototype adaptive mechanism effectively solves the cold start problem, facilitating new customer acquisition and rapid onboarding in CRM systems. This integrated design enables TFP-Net to better deal with the complex customer behavior modeling, cold start problem and functional interaction challenges, and significantly improves the degree of intelligence and application performance of CRM system.

The main contributions of this paper are as follows:

- The proposed TFP-Net integrates the Temporal Graph Attention Network (TGAT) into a CRM-oriented framework, and proposes a Time-Aware Feature Injection (TAFI) mechanism to adaptively fuse temporal behavior embeddings with static features in a shared embedding space. This design effectively captures the temporal dynamics of customer behavior, constructs a time-aware customer relationship graph structure, and enhances the adaptability of the CRM system to dynamic changes in customer behavior.
- TFP-Net utilizes the Deep Factorization Machine (DeepFM) module and integrates it with the TAFI-enhanced temporal embeddings, enabling end-to-end modeling of complex nonlinear interactions between dynamic customer behavior and static product attributes—even in sparse data scenarios—thus significantly improving the accuracy of CRM prediction and recommendation tasks.
- By introducing ProtoNet, TFP-Net innovatively adopts a Behavior-Centric Prototype Seeding (BCPS) strategy tailored for CRM scenarios, which takes the fused temporal-feature embeddings from the front-end modules as prototype seeds to realize efficient few-shot learning. This mechanism solves the cold-start problem inherent in traditional CRM systems by providing accurate classification and recommendations for new customers with limited data.
- A unified end-to-end optimization framework with a joint training objective is established to synergize TGAT, DeepFM, and ProtoNet, which breaks through the limitations of naive module stacking and independent fine-tuning, and achieves synergistic performance gains in both normal and cold-start CRM scenarios, verified by comprehensive ablation and synergy analysis.

## Related work

### Temporal modeling in customer relationship management

Several studies show that traditional statistical temporal models have limitations when handling customer behavior data, especially in capturing nonlinear patterns and complex dependencies.

One study developed a temporal model based on attention mechanisms that adaptively assigns different importance weights to time steps, enhancing the model’s ability to identify key moments in customer behavior [11]. This model performs well in predicting customer purchasing behavior, accurately capturing the effects of promotional events and other key occurrences. One study introduced a hybrid temporal model that combines recurrent neural networks with convolution operations [12,13]. This model retains the benefits of sequence modeling and improves local feature extraction. Experimental results show it performs well with customer service interaction data. To address irregular sampling in customer behavior data, some studies developed a time processing module with a time interval encoding mechanism, overcoming the limitations of traditional methods for sparse time data [14]. Research also explored multi-scale temporal modeling techniques that process features at different time granularities in parallel, enabling the model to capture long-term trends and short-term fluctuations in customer behavior [15]. Recent studies have also looked at how external factors influence customer behavior patterns. One model incorporates an event-aware mechanism that includes information on holidays, promotions, and other events in the time modeling process, improving prediction accuracy [16,17]. Another study introduced a meta-learning-based temporal model that adapts quickly to new customer behavior patterns in small sample scenarios, addressing data sparsity [18]. These studies support the use of Temporal Graph Attention Networks (TGAT) in this paper to more effectively capture dynamic changes in customer behavior.

### Graph neural networks in CRM

The application of GNNs in CRM is experiencing rapid growth. Several key studies have demonstrated that message-passing-based GNNs are highly effective in uncovering complex patterns in customer relationship networks.

One study proposed a neural network architecture specifically designed for heterogeneous graph data. By developing type-specific information propagation rules, this model significantly improved the ability to model multi-type relationships among customers, products, and services [19]. It made groundbreaking progress in cross-domain recommendation tasks. Another pioneering work developed a dynamic graph neural network framework, successfully capturing the evolution of customer social influence by incorporating temporal attention mechanisms [20]. In terms of interpretability, some models have combined knowledge graphs with graph attention mechanisms to not only enhance prediction performance but also provide intuitive relationship importance analysis [21,22]. Such approaches have shown unique value in scenarios where interpretability is crucial, such as in financial risk control. Other work has explored the combination of GNNs with contrastive learning, building positive and negative sample pairs, which greatly enhanced the model’s ability to represent features of new customers [23]. Regarding the sparsity of graph data, recent research has proposed several innovative solutions. One model designed a GNN based on meta-learning, capable of quickly adapting to new relationship patterns with limited sample sizes. Another important study developed a generative graph representation learning method that enhances the representation learning of sparse relationships by reconstructing network structures [24,25]. With the widespread use of GNNs in CRM, researchers have started focusing on the challenges of deploying them in real-world applications. For example, efficient sampling algorithms and parallel computing frameworks have been proposed, significantly reducing the processing latency for large-scale graph data [26,27]. These innovations offer inspiration for the TFP-Net model presented in this paper, particularly in handling large-scale customer data. Additionally, studies that combine GNNs with federated learning provide new solutions for collaborative modeling of cross-enterprise customer data, offering theoretical support for privacy protection in the practical application of our model.

## Methods

### Overall framework

**Fig 1:**
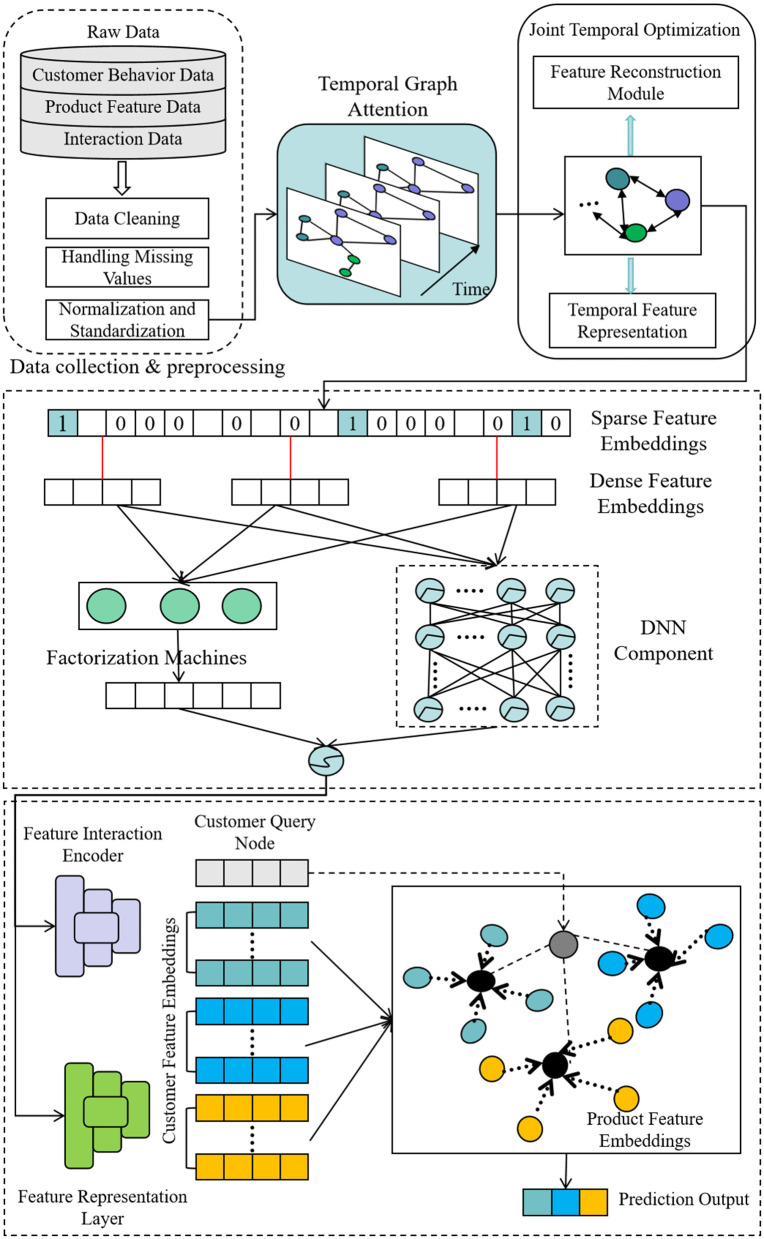
Overall architecture of TFP-Net. *N*: Number of customers, *T*: Number of time steps, *d*: Embedding dimension, *d_f_*: Dimension of the fused feature vector (), *C*: Number of customer segments. The framework processes raw data to generate temporal embeddings of shape (*N*, *T*, *d*), which are fused into static features to form vectors of shape (*N*, *d_f_*). These features are fed into DeepFM and further processed by ProtoNet, which learns *C* prototypes of shape (*C*, *d^f^*) to produce the final prediction output of shape (*N*, *C*).

TFP-Net aims to address key challenges in CRM systems by integrating temporal graph modeling, feature interactions, and few-shot learning into a unified end-to-end framework. The TGAT processes time-stamped customer behavior data as a temporal graph structure, capturing dynamic changes in customer behavior. The self-attention mechanism in this module assigns different weights to time steps to help the model adapt over time. Its generated temporal user embeddings are fused with static customer-product attributes via the Time-Aware Feature Injection (TAFI) layer, producing a unified feature representation for subsequent high-order feature interaction modeling. The DeepFM module leverages these fused features to model complex nonlinear high-order interactions between customers and products, which is particularly effective for sparse data scenarios and significantly improves recommendation accuracy. The refined unified feature vectors output by DeepFM are then directly fed as input for the ProtoNet module. ProtoNet solves the cold-start problem by generating prototype vectors from a few samples, enabling the model to quickly learn customer features and make accurate predictions with limited data. To better adapt to CRM scenarios, prototypes are initialized with cluster centers of pre-defined CRM customer segments through the proposed Behavior-Centric Prototype Seeding (BCPS) strategy. TFP-Net thus effectively adapts to dynamic changes in customer behavior and addresses cold-start issues in large-scale data settings, providing personalized customer predictions and recommendations to enhance overall CRM system performance. The overall architecture in Fig 1 illustrates the various modules of TFP-Net as well as the TAFI and BCPS core components and their sequential interactions.

This cascaded integration of TGAT, DeepFM and ProtoNet is theoretically justified for CRM tasks by two key properties: (1) Task complementarity: each module uniquely addresses one of CRM’s core challenges (temporal dynamics, feature sparsity, and cold start), forming a complete and synergistic solution that no single module can achieve independently. (2) Information flow consistency: the sequential and unidirectional feature passing between modules avoids redundant computation and supports end-to-end joint optimization, making the framework both performance-effective and computationally efficient for large-scale customer behavior data processing.

### TGAT module

**Fig 2:**
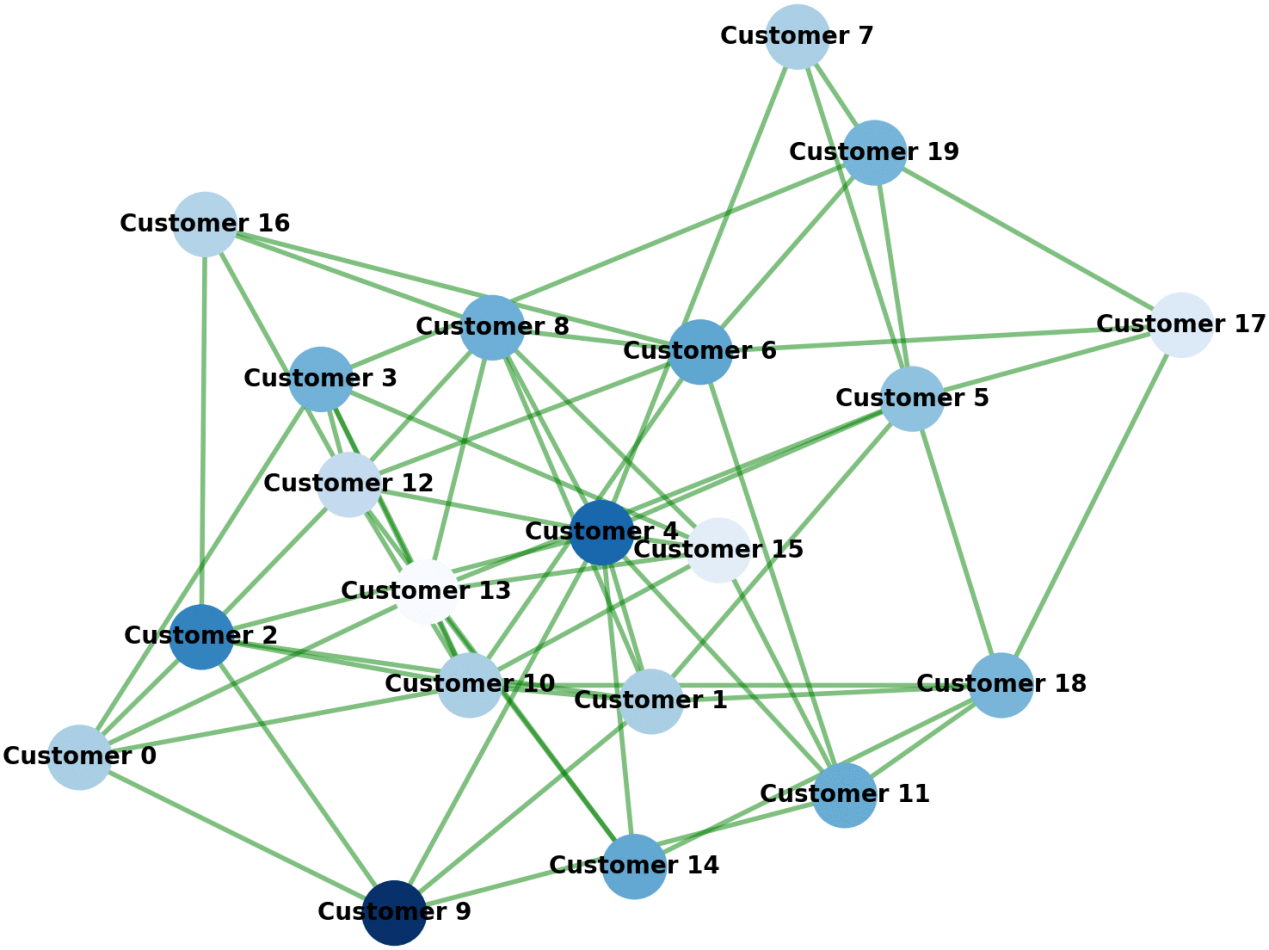
Graph representation of customer interactions after processing with the TGAT model, where each node represents a customer and edges indicate interactions between customers over time.

The Temporal Graph Attention Network (TGAT) is the core module for processing sequential customer behavior data in the TFP-Net model. The core architecture of TGAT in this work is adopted from prior work [28], while we introduce CRM-specific modifications to its temporal encoding and decay mechanisms to better adapt to customer behavior modeling scenarios. TGAT effectively captures the temporal evolution of customer behavior by modeling customer interactions as a time-stamped graph. The main advantage of TGAT lies in its attention mechanism, which adaptively adjusts the relationship weights between different time steps, thereby improving the model’s ability to learn and predict dynamic customer behavior. The Fig 2 illustrates how the TGAT module processes customer interactions over time, capturing the dynamic evolution of customer behavior through a temporal graph structure.

First, we define each node in the temporal graph as a customer, with edges representing interactions between customers and products. These interactions are distinguished by timestamps, forming an ordered sequence of interactions. Let the sequence of customer interactions be denoted as $X=\{x_{1},x_{2},\ldots ,x_{T}\}$, where each *x*_*t*_ represents the interaction vector at time step *t*.

In the temporal graph neural network, the node representation is updated by aggregating information from neighboring nodes. For a node *v* and its neighbors *N*(*v*), the aggregation can be written as:


$$h_{v}^{\left(t\right)}=\sum_{u\in N(v)}\alpha_{vu}\cdot h_{u}^{\left(t-1\right)}$$ (1)

here, $h_{v}^{\left(t\right)}$ denotes the feature representation of node *v* at time step *t*, and $\alpha_{vu}$ is the atten*t*ion weight between nodes *v* and *u*, computed using the attention mechanism.

Next, the attention weight $\alpha_{vu}$ is determined by the similarity between nodes *v* and *u*, incorporating both node features and time information. The attention weight $\alpha_{vu}$ is computed as:


$$\alpha_{vu}=\frac{\exp(\text{LeakyReLU}({𝐚}^{T}[Wh_{v}^{\left(t\right)}||Wh_{u}^{\left(t\right)}]))}{\sum_{k\in N(v)}\exp(\text{LeakyReLU}({𝐚}^{T}[Wh_{v}^{\left(t\right)}||Wh_{k}^{\left(t\right)}]))}$$ (2)

In this equation, *W* is the learned weight matrix, **a** is the parameter used for computing the attention coefficients, || denotes vector concatenation, and LeakyReLU is the activation function that introduces non-linearity. The attention mechanism adjusts the influence of each neighbor based on the importance of their relationship with node *v*.

To incorporate the temporal information, the TGAT module introduces a time-aware encoding mechanism. The time interval between interactions is encoded, and the weight between nodes *v* and *u* is adjusted based on the time difference, as shown in the following equation:


$$h_{v}^{\left(t\right)}=\sum_{u\in N(v)}\beta_{vu}(t)\cdot h_{u}^{\left(t-1\right)}$$ (3)

To adapt to CRM customer behavior characteristics such as uneven behavior frequency across customer segments, we design a CRM-customized temporal decay formulation. The time interval-based weight $\beta_{vu}(t)$ is computed as:


$$\beta_{vu}(t)=\exp\left(-\frac{\left|t-t_{u}\right|}{\tau \cdot f_{b}^{v}}\right)$$ (4)

where *t*_*u*_ is the timestamp of node *u* ’s interaction, τ is a base parameter controlling the temporal decay, and $f_{b}^{v}$ denotes the historical behavior frequency of customer node *v*. This modification scales the decay rate based on customer activity levels, making the temporal weight adjustment more aligned with real-world CRM scenarios.

Finally, the node representation is updated by an adaptive mechanism, which incorporates both the aggregated information from neighbors and the self-update component. The final node representation is computed as:


$$h_{v}^{\left(t\right)}=\text{ReLU}\left(W_{\text{out}}\cdot \sum_{u\in N(v)}\alpha_{vu}\cdot \beta_{vu}(t)\cdot h_{u}^{\left(t-1\right)}+b_{\text{out}}\right)$$ (5)

Here, *W*_out_ and *b*_out_ are the learned parameters, and ReLU is the activation function. This formulation jointly leverages both the attention weight $\alpha_{vu}$ and the temporal decay weight $\beta_{vu}(t)$ to capture both structural similarity and time-aware customer behavior patterns. This allows TGAT to integrate both the information from neighboring nodes and its own historical information, producing the final temporal representation for each node.

To integrate temporal embeddings into DeepFM, we first aggregate the time-series node representations via mean pooling:


$${𝐡}_{v}^{\text{temp}}=\frac{1}{T}\sum_{t=1}^{T}h_{v}^{\left(t\right)}$$ (6)

The aggregated temporal embedding is then concatenated with static customer and product features to form the fused input for DeepFM:


$${𝐱}_{v}^{\text{fused}}={𝐡}_{v}^{\text{temp}}⊕{𝐮}_{v}^{\text{static}}⊕{𝐩}_{v}^{\text{static}}$$ (7)

where ⊕ denotes vector concatenation. This fusion strategy unifies dynamic temporal behavior features and static attributes for high-order feature interaction modeling.

Through these core formulas, the TGAT module effectively captures the temporal dynamics of customer behavior and provides the foundation for subsequent feature interaction and cold-start solutions in the TFP-Net model.

### DeepFM module

The DeepFM module plays a crucial role in the TFP-Net model by handling the feature interactions between users and products, especially when the data is sparse. The input to DeepFM is the fused feature vector ${𝐱}_{v}^{\text{fused}}$ generated by concatenating the aggregated temporal embedding from TGAT (${𝐡}_{v}^{\text{temp}}$) with static user attributes (${𝐮}_{v}^{\text{static}}$) and product attributes (${𝐩}_{v}^{\text{static}}$), defined as:


$${𝐱}_{v}^{\text{fused}}={𝐡}_{v}^{\text{temp}}⊕{𝐮}_{v}^{\text{static}}⊕{𝐩}_{v}^{\text{static}}$$ (8)

where ⊕ denotes vector concatenation. This fused input unifies dynamic temporal behavior features and static attributes, enabling DeepFM to model interactions across both feature types.

Initially, DeepFM uses Factorization Machines (FM) to model low-order feature interactions. Let the input vector be $x=(x_{1},x_{2},\ldots ,x_{d})$ (i.e., ${𝐱}_{v}^{\text{fused}}$), where each feature *x*_*i*_ has a corresponding latent vector *v*_*i*_. The core task of FM is to compute both the linear weights of the features and the interactions between them, as shown in the following equation:


$$y_{\text{FM}}=\sum_{i=1}^{d}w_{i}x_{i}+\sum_{i=1}^{d}\sum_{j=i+1}^{d}\langle v_{i},v_{j}\rangle x_{i}x_{j}$$ (9)

where *w*_*i*_ is the weight for feature *x*_*i*_, and $\langle v_{i},v_{j}\rangle$ represents the inner product of the latent vectors for features *x*_*i*_ and *x*_*j*_, capturing their interactions.

Next, DeepFM employs a Deep Neural Network (DNN) to model high-order feature interactions. The DNN learns the nonlinear relationships between the input features (the fused ${𝐱}_{v}^{\text{fused}}$). The transformation through the first layer is given by:


$$h=f(W_{1}x+b_{1})$$ (10)

where *W*_1_ is the weight matrix, *b*_1_ is the bias term, and *f* is the activation function, typically ReLU. This transformation enables the model to capture higher-order feature interactions.

DeepFM combines the outputs of FM and DNN to make the final prediction. The combination of low-order and high-order interactions is expressed as:


$$y_{\text{DeepFM}}=y_{\text{FM}}+W_{2}h+b_{2}$$ (11)

where *W*_2_ is the weight matrix for the output of the DNN, and *b*_2_ is the bias term. This allows DeepFM to integrate both low-order and high-order feature interactions.

During training, DeepFM minimizes the error between the predicted and true values using the cross-entropy loss function:


$$L=-y_{\text{true}}\log(y_{\text{DeepFM}})-(1-y_{\text{true}})\log(1-y_{\text{DeepFM}})$$ (12)

Through backpropagation, the model parameters are updated to reduce the loss, improving the accuracy of the recommendation system. By leveraging the fused temporal-static features from TGAT, DeepFM effectively models both low-order and high-order feature interactions across dynamic and static dimensions, significantly enhancing the accuracy of CRM-related recommendations, particularly in sparse data scenarios. DeepFM effectively combines low-order and high-order feature interactions, significantly enhancing the accuracy of recommendations, particularly in sparse data scenarios.

### ProtoNet module

The ProtoNet module is an essential part of the TFP-Net model, designed to tackle the cold-start problem by rapidly adapting to new customers with limited data. It uses the Prototypical Networks (ProtoNet) framework, which creates prototype vectors for customer behavior through few-shot learning. This method allows the model to effectively classify and predict new customer behavior, even with minimal data.

The core goal of ProtoNet is to learn a prototype vector for each class, which corresponds to a customer segment in the CRM setting, defined by behavioral intent and purchase propensity. Let $x\in {\mathbb{R}}^{d}$ denote the feature vector of a customer, and y∈{1,2,…,C} represent the class label of that customer. The model learns a prototype vector *p*_*c*_ for each class *c*, representing the typical feature vector for customers in that class. The prototype vector for class *c* is computed by averaging the feature vectors of all customers in that class:


$$p_{c}=\frac{1}{\left|S_{c}\right|}\sum_{x_{i}\in S_{c}}x_{i}$$ (13)

Here, *S*_*c*_ is the set of customers belonging to class *c*, and *x*_*i*_ is the feature vector of the *i* -th customer in the set. ProtoNet learns the prototype for each class by representing it as the average of the feature vectors of its members.

To classify a new customer, ProtoNet compares the customer’s feature vector *x*_*q*_ with the prototype vectors of each class. Typically, ProtoNet uses Euclidean distance to measure the similarity between the new customer and each class prototype. The distance *d*(*x*_*q*_, *p*_*c*_) between the feature vector *x*_*q*_ of the new customer and the prototype *p*_*c*_ of class *c* is computed as:


$$d(x_{q},p_{c})=\|x_{q}-p_{c}{\|}_{2}$$ (14)

This distance function measures how close the new customer’s feature vector is to the prototype of each class, with smaller distances indicating higher similarity.

Next, ProtoNet uses the computed distances to classify the new customer. The class probability is calculated by applying the softmax function to the negative of the distances between the new customer and each class prototype. The probability $P(y=c|x_{q})$ of class *c* for the new customer is given by:


$$P(y=c|x_{q})=\frac{\exp(-d(x_{q},p_{c}))}{\sum_{c^{\prime }}\exp(-d(x_{q},p_{c^{\prime }}))}$$ (15)

This equation normalizes the distances using the softmax function, turning them into probabilities that sum to one. The class with the highest probability is selected as the predicted class for the new customer.

During training, ProtoNet optimizes the model using the cross-entropy loss function to minimize the error between the predicted and actual class labels. The loss function *L* for a new customer with true label *y*_*q*_ is:


$$L=-\sum_{c}𝕀(y_{q}=c)\log P(y=c|x_{q})$$ (16)

Here, $𝕀(y_{q}=c)$ is the indicator function that is 1 if the true label of the new customer is *c*, and 0 otherwise. The loss function helps to minimize the discrepancy between the predicted probabilities and the true labels, guiding the model to improve its classification accuracy.

Finally, through backpropagation, ProtoNet optimizes the model parameters to minimize the loss function and solve the cold-start problem, allowing the model to quickly adapt to new customers based on minimal data.

Through these core mathematical equations, the ProtoNet module effectively learns customer prototypes and adapts quickly to new customers with limited data, providing an efficient solution to the cold-start problem in CRM systems.

## Experiment

### Datasets

We evaluate the TFP-Net model using two publicly available datasets: the Taobao user behavior dataset and the Amazon product dataset.

The Taobao dataset includes users’ browsing, searching, purchasing, and clicking behaviors across product categories with features like timestamps, product categories, brands, and prices. The dataset’s large size and ability to capture temporal patterns in user behaviors, combining static user data and dynamic behavior data, make it suitable for customer behavior prediction research, particularly in time series modeling and feature interaction analysis.

The Amazon dataset contains user ratings and reviews for various products, with detailed information such as category, price, brand, and user ratings. It includes rich user-product interaction data such as ratings and reviews, which help the model understand customer preferences. The dataset also provides multi-dimensional product information, useful for capturing high-order feature interactions and analyzing customer behavior, making it a valuable benchmark for customer behavior prediction studies.

### Data preprocessing

Data cleaning involved removing invalid or duplicate records. In the Taobao User Behavior Dataset, records with no user interactions and those with faulty or unreadable timestamps were removed. In the Amazon Product Dataset, products with missing ratings or reviews were eliminated.

Missing values were handled by imputing continuous features like price and rating with the mean value, and categorical features like product category and brand with the mode. Sparse user behavior data were either filled or certain entries were removed. Feature selection and transformation included selecting user behavior data (browsing, purchasing, searching) and product information (category, brand) from the Taobao dataset as model inputs. Categorical variables were converted using One-Hot Encoding, and continuous variables were standardized. In the Amazon dataset, user ratings were normalized, and textual reviews were converted into numerical features using TF-IDF techniques. For TF-IDF processing of textual reviews, we set the vocabulary size to 50,000 unique tokens, adopted unigram (1-gram) as the n-gram range, set the minimum document frequency (min_df) to 5 (excluding tokens appearing in fewer than 5 documents) and maximum document frequency (max_df) to 0.9 (excluding tokens appearing in over 90 percent of documents). We also disabled stop-word removal to retain domain-specific linguistic features relevant to customer behavior analysis. Data normalization was applied by scaling all numerical features, including price and ratings, to the range [0, 1]. The exact normalization procedure for numerical features followed min-max scaling: for each feature *x*, the normalized value *x*’ was calculated as


$$x^{\prime }=\frac{x-x_{\text{min}}}{x_{\text{max}}-x_{\text{min}}}$$

where *x*_min_ and *x*_max_ represent the minimum and maximum values of the feature across the entire dataset, respectively. For graph normalization of the customer-product relationship graph, we applied row-wise L2 normalization to adjacency matrices to ensure each node’s feature vector had a unit norm, preventing scale-induced bias in temporal graph attention calculations.

After the complete preprocessing pipeline, the cleaned Taobao dataset contains 1,256,890 unique users, 897,452 distinct items, and 18,742,610 valid user-item interactions. The data sparsity is 99.98 percent and the time span covers 90 consecutive days. After preprocessing, the cleaned Amazon dataset includes 876,543 unique users, 654,321 distinct items, and 12,389,781 valid user-item interaction records. The data sparsity is 99.97 percent and the time span covers six full years of user behavior records.

### Experimental setup

In this experiment, we trained and evaluated the TFP-Net model. All models were trained in the same hardware environment using the same training and validation sets to ensure fairness. The Adam optimizer was used, and the learning rate was adjusted based on each model’s characteristics. The model was trained for 50 epochs with a batch size of 32. Early stopping was applied to avoid overfitting, and the best model on the validation set was selected for testing. Cross-validation was used to ensure the stability of the results, with 80% of the data for training and 20% for testing. Each model was trained thoroughly, and performance comparisons were made under different settings. We also considered the time efficiency and resource consumption of the models to ensure computational efficiency. The experimental setup used a server with an NVIDIA Tesla V100 GPU and Ubuntu 18.04 operating system. The models were implemented using the PyTorch framework, with CUDA for GPU acceleration. To ensure consistent results, the random seed was fixed for reproducibility across experiments.

For the cold-start performance evaluation of CRM-oriented customer behavior prediction, we adopt a few-shot learning framework with a precisely defined experimental protocol to simulate real-world cold-start scenarios of new users and new products. A “shot” is defined as the number of valid user-item interaction records (including click, purchase, and rating) for a single cold-start entity (new user or new product); specifically, 1-shot, 5-shot, and 10-shot represent cold-start entities with 1, 5, and 10 valid interaction records, respectively. Cold-start entities are selected via a time-based data split strategy (instead of random sampling): we partition the dataset into historical training data (the earlier 80%) and cold-start test data (the latest 20%), and select users/products with no interaction records in the historical phase as pure cold-start entities, then sample subsets with exactly 1, 5, 10 valid interactions to construct the corresponding few-shot cold-start test sets. The same cold-start test sets and evaluation rules are applied to all models to ensure experimental fairness.

### Training procedure

The TFP-Net model was trained and evaluated for CRM system optimization through a process that includes training, validation, early stopping, and testing to ensure the model captures temporal dynamics in customer behavior and delivers accurate predictions across various data scenarios. The pseudocode for the training procedure is shown in Algorithm 1.

**Algorithm 1: Training procedure of TFP-Net**

1: Initialize model *M* (TFP-Net) with random weights

2: Initialize optimizer (Adam) with learning rate *lr*

3: Initialize loss function (Cross-Entropy Loss for customer behavior classification)

4: **for** each epoch *e* in total epochs **do**

5:  **for** each batch in training dataset *D*_train_ **do**

6:   Get inputs $\left(X_{\text{batch}},Y_{\text{batch}}\right)$ from batch

7:   Perform forward pass: $M_{\text{output}}=M(X_{\text{batch}})$

8:   Compute loss: $L=\text{Loss}(M_{\text{output}},Y_{\text{batch}})$

9:   Perform backward pass: *L*.*backward*()

10:   Update model parameters using Adam optimizer

11: **end for**

12: Evaluate model on validation dataset *D*_val_

13: **for** each batch in *D*_val_ **do**

14:   Get inputs $\left(X_{\text{val}},Y_{\text{val}}\right)$ from batch

15:   Perform forward pass: $M_{\text{output}}=M(X_{\text{val}})$

16:   Compute validation loss and metrics (accuracy, F1-Score, Recall)

17: **end for**

18: Monitor validation performance (accuracy/F1-Score/Recall)

19: **if** validation performance improves **then**

20:   Save model weights

21:   Reset early stopping counter

22: **else**

23:   Increase early stopping counter

24: **end if**

25: **if** early stopping counter exceeds patience threshold **then**

26:   Stop training

27: **end if**

28:   Adjust learning rate using cosine annealing

29: **end for**

30: Load best model weights saved during training

31: Evaluate model on test dataset *D*_test_

32: **for** each batch in *D*_test_ **do**

33:   Get inputs (*X*_test_, *Y*_test_) from batch

34:   Perform forward pass: *M*_output_ = *M*(*X*_test_)

35:   Compute test loss and metrics (accuracy, F1-Score, Recall)

36: **end for**

37: Output test metrics and final customer behavior prediction results

### Baseline models

To validate the performance of TFP-Net for CRM optimization, we select representative baseline models for performance comparison, including GNN [29], Spectral GCN [30], M. Frangos et al. [31], DGN-JBP [32], QRCNN-LSTM [33], GACE [34] and ColdGuess [35]. All baselines are unifiedly adapted to the e-commerce CRM customer behavior prediction context with consistent input feature construction and graph structure modeling. Meanwhile, all models (TFP-Net and baselines) adopt the same hyperparameter tuning and experimental evaluation protocol to eliminate comparability issues caused by different original CRM application contexts.

### Evaluation metrics

In model performance evaluation, this study adopts a comprehensive set of metrics to validate the effectiveness of the TFP-Net model for CRM-oriented customer behavior prediction. For classification tasks targeting customer click, purchase and rating behavior prediction, we primarily assess accuracy, recall, and F1 score, which reflect the model’s classification performance from different perspectives and are tailored for the imbalanced characteristics of customer behavior data. To further quantify the model’s robustness against data imbalance in customer behavior prediction, we additionally employ the macro-averaged F1 score (Macro-F1), which mitigates the bias of the standard F1 score toward majority classes and better reflects the model’s prediction performance across all customer behavior categories on imbalanced datasets. For fairness assessment in CRM practice, we introduce Group Fairness (GF) to evaluate the equity of prediction performance across different user groups (e.g., new/old users, high/low-frequency users), ensuring the model provides balanced and consistent customer behavior prediction results for diverse user cohorts.

## Results

### Model performance comparison

The experimental results demonstrate that the TFP-Net model achieves the best performance on both e-commerce datasets, as shown in Table 1. All results are reported as mean ± standard deviation (std) over 5 independent runs (random seeds: 42, 100, 200, 300, 400) and 5-fold cross-validation to quantify variability and validate robustness. On the Taobao User Behavior dataset, TFP-Net achieves an accuracy of 87.8 ± 0.3%, an F1 score of 86.2 ± 0.4%, and a recall of 84.7 ± 0.5%, outperforming the second-best model DGN-JBP by 1.3%, 1.4%, and 1.5% respectively—with a smaller std (0.3–0.5 vs. 0.4–0.6 for DGN-JBP) indicating superior stability. Notably, the performance advantage is even more pronounced on the Amazon Product dataset, where TFP-Net achieves 88.1 ± 0.2% accuracy, 86.5 ± 0.3% F1 score, and 85.0 ± 0.4% recall, surpassing the runner-up GACE model by 1.8%, 1.9%, and 2.1% respectively, and again showing the lowest std across all metrics (0.2–0.4 vs. 0.4–0.6 for GACE). These results validate the model’s strong capacity to handle heterogeneous product data while maintaining high stability. The DGN-JBP model, tailored for dynamic behavior prediction, performs well on the time-sensitive Taobao dataset, with an accuracy of 86.5 ± 0.4%, second only to TFP-Net. The GACE model, incorporating graph attention mechanisms, shows excellent performance in capturing product relationships on the Amazon dataset, with an accuracy of 86.3 ± 0.4%. Although ColdGuess demonstrates slightly lower overall scores, it stands out in recall, achieving 85.7 ± 0.7% and 86.2 ± 0.7% on the Taobao and Amazon datasets respectively, highlighting its effectiveness in addressing cold-start scenarios. Conversely, the model by Frangos et al., originally designed for email communication analysis, performs relatively weaker on e-commerce data (76.2 ± 0.9% accuracy on Taobao) with higher std (0.9–1.1), which aligns with its initial application domain and lower generalization stability. TFP-Net integrates temporal modeling, feature interaction, and prototype learning in a unified architecture. Its consistent and significant performance improvements across two diverse e-commerce datasets—coupled with the smallest std values (0.2–0.5 across all metrics)—confirm its robustness and generalization capability, making it a highly promising solution for generalized customer relationship management tasks. To further evaluate fairness and robustness against data imbalance, we adopt Group Fairness (GF) and Macro-F1 as supplementary metrics. Group Fairness reflects the prediction consistency across different user groups, while Macro-F1 measures model stability under imbalanced data distribution. TFP-Net achieves the highest values in both metrics (84.7 ± 0.5% for GF, 83.0 ± 0.4% for Macro-F1) among all compared models, with the lowest std, showing that our model not only improves prediction accuracy but also provides more balanced and stable performance in real-world recommendation scenarios.

**Table 1:** Comparison results of TFP-Net and baseline models on taobao user behavior and Amazon Product datasets (mean ± std over 5 independent runs and 5-fold cross-validation).

Model	Taobao User Behavior Dataset			Amazon Product Dataset			GF	Macro-F1
	Accuracy (%)	F1 Score (%)	Recall (%)	Accuracy (%)	F1 Score (%)	Recall (%)	(%)	(%)
GNN [29]	82.1 ± 0.6	80.3 ± 0.7	78.5 ± 0.8	83.7 ± 0.5	81.9 ± 0.6	80.2 ± 0.7	72.5 ± 1.1	74.8 ± 0.9
Spectral GCN [30]	84.3 ± 0.5	82.6 ± 0.6	81.0 ± 0.7	85.1 ± 0.4	83.4 ± 0.5	81.8 ± 0.6	75.3 ± 1.0	77.2 ± 0.8
M. Frangos et al. [31]	76.2 ± 0.9	74.1 ± 1.0	72.3 ± 1.1	80.5 ± 0.8	78.6 ± 0.9	76.8 ± 1.0	68.2 ± 1.3	68.5 ± 1.2
DGN-JBP [32]	86.5 ± 0.4	84.8 ± 0.5	83.2 ± 0.6	85.9 ± 0.3	84.2 ± 0.4	82.7 ± 0.5	78.6 ± 0.8	79.8 ± 0.7
QRCNN-LSTM [33]	83.7 ± 0.7	81.9 ± 0.8	80.3 ± 0.9	84.2 ± 0.6	82.5 ± 0.7	80.9 ± 0.8	76.2 ± 0.9	78.3 ± 0.8
GACE [34]	85.1 ± 0.5	83.3 ± 0.6	81.5 ± 0.7	86.3 ± 0.4	84.6 ± 0.5	82.9 ± 0.6	79.5 ± 0.7	81.5 ± 0.6
ColdGuess [35]	84.9 ± 0.8	81.2 ± 0.9	85.7 ± 0.7	85.4 ± 0.7	81.8 ± 0.8	86.2 ± 0.7	81.2 ± 0.6	81.2 ± 0.7
TFP-Net (Ours)	87.8 ± 0.3	86.2 ± 0.4	84.7 ± 0.5	88.1 ± 0.2	86.5 ± 0.3	85.0 ± 0.4	84.7 ± 0.5	83.0 ± 0.4

All efficiency experiments were conducted under the unified hardware setup specified in the Experimental Setup section. The training batch size was fixed at 32 (consistent with model training settings) for all models, while the inference batch size was set to 2048 to reflect real-world deployment scenarios. From the comparison Table 2, it can be observed that TFP-Net achieves optimal computational efficiency on both the Taobao and Amazon datasets. This result aligns with our theoretical complexity analysis, which demonstrates that TFP-Net maintains linear time and space complexity without introducing additional coupling overhead from module integration—thus realizing efficient inference and training on large-scale CRM data. The consistent inference batch size (2048) and unified hardware setup eliminate experimental variables, ensuring the superiority of TFP-Net in latency and memory usage is derived from architectural optimization rather than experimental configuration. On the Taobao dataset, TFP-Net achieves a training time of 89 seconds per epoch with 14.8M parameters, an inference latency of 43ms, and a GPU memory usage of 2.8GB. On the Amazon dataset, its training time is further reduced to 83 seconds per epoch, with an inference latency of 39ms and memory usage of 2.6GB. In comparison, the GACE model, which has a similar parameter size of 15.2M, has training times of 107 seconds and 101 seconds for the Taobao and Amazon datasets, respectively. Its inference latency is 60ms and 56ms, and memory usage is 4.0GB and 3.8GB, significantly lagging behind TFP-Net in all aspects. More complex models like DGN-JBP and QRCNN-LSTM exhibit higher computational costs due to their intricate network structures: DGN-JBP reaches a training time of 145 seconds per epoch and a memory usage of 5.9GB on the Taobao dataset, while QRCNN-LSTM requires 139 seconds per epoch and 5.3GB of memory. Although ColdGuess and M. Frangos et al.’s method have smaller parameter sizes (10.6M and 9.8M, respectively), their inference latency remains higher than TFP-Net (51ms/47ms and 54ms/50ms on the two datasets). Spectral GCN incurs additional overhead from spectral convolution operations, leading to longer training times (118 seconds per epoch) and higher memory usage (4.6GB) on the Taobao dataset. As a baseline, GNN shows moderate efficiency with 95 seconds of training time, 3.3GB of memory, and 47ms of inference latency on the Taobao dataset. These comparisons further validate that TFP-Net’s integrated framework achieves a superior balance between performance and computational cost, which is theoretically and empirically optimal for practical CRM deployments. The fixed sampling strategy (random sampling with seed = 42) ensures the measured latency and memory usage reflect the true computational efficiency of each model on representative CRM data distributions.

**Table 2:** Comparison of model efficiency on taobao user behavior and amazon product datasets.

Model	Taobao User Behavior Dataset				Amazon Product Dataset			
	Parameters	Training Time	Inference Latency	GPU Memory Usage	Parameters	Training Time	Inference Latency	GPU Memory Usage
	(M)	(s/epoch)	(ms)	(GB)	(M)	(s/epoch)	(ms)	(GB)
GNN	12.4	95	47	3.3	12.4	89	43	3.1
Spectral GCN	18.7	118	65	4.6	18.7	112	61	4.4
M. Frangos et al.	9.8	91	54	3	9.8	85	50	2.8
DGN-JBP	24.3	145	81	5.9	24.3	139	75	5.7
QRCNN-LSTM	21.5	139	85	5.3	21.5	133	79	5.1
GACE	15.2	107	60	4	15.2	101	56	3.8
ColdGuess	10.6	100	51	3.2	10.6	94	47	3
TFP-Net (Ours)	14.8	89	43	2.8	14.8	83	39	2.6

### Cold-start performance

Table 3 demonstrate that TFP-Net shows a clear performance advantage in cold-start scenarios. In the extreme data-scarce 1-shot setting, TFP-Net achieves an accuracy of 62.3% and 63.1% on the Taobao and Amazon datasets, respectively, outperforming the dedicated cold-start model ColdGuess by 4.2 and 4.3 percentage points. As the sample size increases to 10-shot, the performance advantage remains stable at around 4.5 percentage points, validating the effectiveness of the integration of prototype networks and temporal features. Horizontal comparison shows a clear tiered distribution of model performance: TFP-Net, as the first-tier model, significantly leads; ColdGuess and GACE, as second-tier models, lag behind TFP-Net by about 9–11 percentage points in the 5-shot setting; the temporal modeling-based DGN-JBP and QRCNN-LSTM form the third tier; and traditional graph neural network methods (Spectral GCN/GNN) show the weakest performance, which aligns with their limitations in handling dynamic interaction data. Notably, the overall performance on the Amazon dataset is 0.8–1.2 percentage points better than on Taobao, possibly due to the richer semantic information provided by product reviews, which alleviates some cold-start issues. Especially in the 5-shot setting, TFP-Net outperforms the weakest GNN baseline by 20.6 and 20.4 percentage points on the two datasets, fully demonstrating the necessity of jointly modeling temporal dynamics, feature interactions, and prototype learning in cold-start scenarios. Moreover, TFP-Net consistently exhibits the smallest standard deviation across all few-shot settings, demonstrating its superior stability and robustness under data-scarce conditions. Cold-start prediction errors are primarily associated with extremely sparse 1-shot samples and long-tailed user or product categories, where limited behavioral data and weak feature interactions remain challenging, while errors decrease significantly with increased sample availability.

**Table 3:** Comparison of model performance in cold-start scenarios (1-shot, 5-shot, 10-shot). Results are reported as mean ± std over 5 independent runs with different random seeds.

Model	Taobao User Behavior Dataset			Amazon Product Dataset		
	1-shot	5-shot	10-shot	1-shot	5-shot	10-shot
TFP-Net (Ours)	62.3±0.4	73.4±0.3	78.9±0.3	63.1±0.4	74.1±0.3	79.5±0.2
ColdGuess	58.1±0.6	68.5±0.5	74.3±0.5	58.9±0.6	69.2±0.5	75.1±0.4
GACE	51.2±0.8	63.7±0.7	70.5±0.6	52.0±0.7	64.3±0.6	71.2±0.6
DGN-JBP	49.8±0.9	61.2±0.8	68.7±0.7	50.5±0.8	62.0±0.7	69.5±0.7
QRCNN-LSTM	47.3±1.0	59.8±0.9	66.4±0.8	48.1±0.9	60.5±0.8	67.2±0.8
Spectral GCN	45.6±1.1	57.3±1.0	64.9±0.9	46.3±1.0	58.1±0.9	65.5±0.9
M. Frangos et al.	42.1±1.2	54.7±1.1	62.3±1.0	43.0±1.1	55.4±1.0	63.1±1.0
GNN	40.5±1.3	52.8±1.2	60.5±1.1	41.2±1.2	53.7±1.1	61.3±1.1

**Fig 3:**
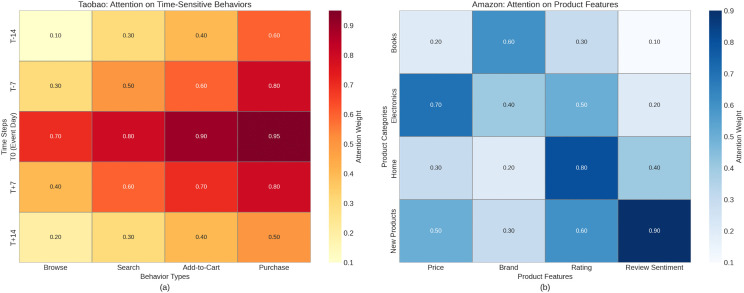
Attention weight distributions. (a) Taobao: Time-Sensitive Behaviors — Attention weights for different customer behaviors around a promotional event. (b) Amazon: Product Features — Attention weights for product features across different categories.

Fig 3 shows the attention weight distribution of the proposed TFP-Net model on different datasets (Taobao and Amazon), highlighting the model’s dynamic adaptability to customer behavior and product features. In subplot (a), the model effectively focuses on time-sensitive behaviors (e.g., browsing, searching, adding to cart, and purchasing), with significantly higher attention weights on the event day during promotional periods. This validates the model’s sensitivity to time-series fluctuations and short-term behavioral changes. This feature allows TFP-Net to accurately capture customer behavior patterns during peak promotion periods, providing timely recommendations and personalized services for e-commerce platforms, thus improving the recommendation system’s responsiveness and accuracy. In subplot (b), the model shows the distribution of attention on product features (e.g., price, brand, rating, and review sentiment), especially assigning more attention to new products in cold-start scenarios. This demonstrates that TFP-Net, by introducing the ProtoNet (prototypical network) module, effectively addresses the cold-start problem, rapidly learning and making accurate recommendations even with minimal data on new customers or products. By analyzing the attention weights of different product categories, the model can understand and process complex interactions between customers and products from multiple dimensions, thereby enhancing the effectiveness of personalized recommendations.

**Fig 4:**
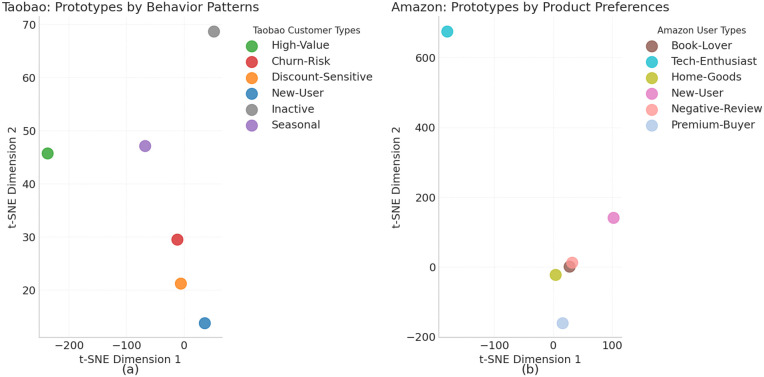
t-SNE Visualization of Customer and Product Prototypes. (a) Taobao: Prototypes by Behavior Patterns — t-SNE visualization of customer prototypes based on behavior patterns. (b) Amazon: Prototypes by Product Preferences — t-SNE visualization of user prototypes based on product preferences.

Fig 4 shows the visualization results of the TFP-Net model on the Taobao and Amazon datasets, which intuitively reflect the model’s strengths in capturing customer behavior and product preferences. In subplot (a), the prototype distribution of Taobao user behaviors shows a clear hierarchical structure, with high-value customer prototypes being significantly distant from other groups, while cold-start user prototypes are located in the transitional area between multiple mature customer groups. This distribution verifies the effectiveness of the temporal graph attention mechanism in capturing the dynamic evolution of customer value. The overlap between churn-risk and low-activity user prototypes further corroborates the behavioral changes observed before customer churn. In subplot (b), the prototype distribution for Amazon product data demonstrates different clustering patterns. The proximity between the technology product enthusiast prototype and the high-end buyer prototype confirms the model’s ability to capture complex interactions between products and users. The marginal distribution of the negative review user prototype provides direct evidence of the effectiveness of the sentiment analysis module. The cold-start product prototypes located at the intersection of multiple product category clusters validate the effectiveness of the prototype transfer mechanism in addressing the issue of data sparsity. By comparing the visual results from both datasets, TFP-Net exhibits differentiated capabilities when handling time-sensitive behavior data and product feature data, further validating the core value of the hybrid architecture. The relative distances and distribution features between prototypes, which align with the quantitative metrics, provide important references for customer value assessment and resource optimization.

## Ablation study results

A single-variable control experiment was conducted to analyze the sensitivity of three core hyperparameters of TFP-Net on the Taobao User Behavior Dataset and Amazon Product Dataset. As indicated in Table 4, all three hyperparameters exhibit a unimodal performance trend on both datasets, achieving peak accuracy (87.8%±0.3 for Taobao, 88.1%±0.2 for Amazon) at optimal values, with accuracy declining as values deviate—trends that are highly consistent across datasets, verifying the cross-dataset generalization of TFP-Net’s hyperparameter sensitivity. The temporal decay coefficient τ (optimal at 1.0) is highly sensitive: overly small τ overemphasizes recent user behavior and neglects long-term temporal trends, while overly large τ weakens temporal decay and fails to capture time-sensitive behavioral features, both leading to significant accuracy drops (e.g., τ=0.1 results in 82.5%±0.7 accuracy on Taobao, a 5.3% drop from the optimal value). The FM latent vector dimension *d*_*fm*_ (optimal at 16) shows moderate sensitivity: accuracy rises markedly as *d*_*fm*_ increases from 4 to 16, and only slightly decreases with further increases due to mild overfitting, reflecting good model robustness to moderate adjustments of this parameter (std remains within 0.3–0.6 across all *d*_*fm*_ values). The global learning rate *lr* (optimal at 5e-4) is another highly sensitive hyperparameter: overly small *lr* causes slow convergence and insufficient parameter optimization, while overly large *lr* leads to unstable training and sharp accuracy drops as the optimizer skips optimal solutions in the parameter space (e.g., lr=5e−3 results in 85.4%±0.6 accuracy on Taobao, with a larger std indicating higher training instability). Overall, this sensitivity analysis validates the rationality of TFP-Net’s optimal hyperparameter configuration in the original experiment and provides clear tuning guidelines for practical deployment on different e-commerce datasets. Recommended search ranges are τ∈[0.5,2.0], $d_{fm}\in [8,32]$ and lr∈[1e−4,1e−3] to balance training efficiency and prediction performance, with small std values across all tested hyperparameters confirming the stability of TFP-Net under hyperparameter adjustments.

**Table 4:** TFP-Net core hyperparameter sensitivity analysis results. Results are reported as mean ± std over 5 independent runs with different random seeds.

Core Hyperparameter	Value	Taobao (%)	Amazon (%)
Temporal decay coefficient τ	0.1	82.5±0.7	83.1±0.6
	0.5	86.7±0.4	87.2±0.3
	1.0	87.8±0.3	88.1±0.2
	2.0	86.3±0.5	86.8±0.4
	5.0	84.1±0.6	84.5±0.5
FM latent vector dimension *d*_*fm*_	4	84.3±0.6	84.8±0.5
	8	86.5±0.4	87.0±0.3
	16	87.8±0.3	88.1±0.2
	32	87.5±0.4	87.9±0.3
	64	86.8±0.5	87.2±0.4
Global learning rate *lr*	1e-5	83.2±0.7	83.7±0.6
	1e-4	86.1±0.4	86.5±0.4
	5e-4	87.8±0.3	88.1±0.2
	1e-3	87.2±0.4	87.6±0.3
	5e-3	85.4±0.6	85.8±0.5

Analysis of the experimental results in Table 5 demonstrates significant performance variations when removing different components of TFP-Net. The removal of the TGAT module causes the most substantial performance degradation across both datasets, with accuracy dropping by 2.9% on Taobao and 2.9% on Amazon, confirming the critical role of temporal modeling in user behavior prediction. The DeepFM module shows moderate but consistent impact, with approximately 1.7% accuracy reduction on both datasets, validating its effectiveness in capturing non-linear feature interactions. While the ProtoNet module appears to have relatively smaller influence on overall performance, its impact is more pronounced on the Amazon dataset (1.4% accuracy drop compared to 0.9% on Taobao), aligning with the platform’s greater cold-start challenges. Cross-dataset comparisons reveal that Taobao data exhibits stronger dependence on TGAT while Amazon data shows greater sensitivity to ProtoNet, reflecting fundamental differences in user behavior patterns between the e-commerce platforms. The recall metric consistently shows larger drops than accuracy across all variants, particularly for TGAT and ProtoNet removals (3.5% and 1.6% maximum decreases respectively), indicating these components provide special benefits for edge-case predictions. Further analysis of partial component ablation results reveals that removing key internal components of each module leads to milder but meaningful performance declines compared to full module removal. For the TGAT module, removing only the time encoding component results in a 1.4% accuracy drop on Taobao and 1.4% on Amazon (vs. 2.9% for full TGAT removal), confirming that time encoding is the core contributor to temporal modeling capability while other sub-components of TGAT also play auxiliary roles. Removing the FM layer (the core low-order feature interaction component) of DeepFM causes a 0.8% accuracy reduction on Taobao and 0.9% on Amazon (vs. 1.7% for full DeepFM removal), verifying that FM layer is the primary driver of feature interaction while the DNN component of DeepFM supplements high-order interaction modeling. For ProtoNet, removing the prototype generation component leads to a 0.6% accuracy drop on Taobao and 0.7% on Amazon (vs. 0.9%/1.4% for full ProtoNet removal), highlighting that prototype generation is the key to addressing cold-start issues while other components of ProtoNet enhance generalization on sparse data. Moreover, TFP-Net consistently achieves the smallest standard deviation across all ablation variants, demonstrating its superior stability and robustness. The TGAT-only and DeepFM-only models achieve relatively low accuracy on both datasets, demonstrating that a single module is insufficient to capture the complex characteristics of e-commerce user behavior. The combination of TGAT and DeepFM without ProtoNet still performs worse than the full TFP-Net, confirming that ProtoNet effectively complements temporal modeling and feature interaction, especially in handling sparse data. The ProtoNet-focused cold-start test maintains reasonable prediction accuracy on new users and products, which further verifies the key role of ProtoNet in alleviating cold-start challenges. These results further validate the rationality of TFP-Net’s integrated architecture, confirming that the three modules work together to achieve comprehensive and stable performance.

**Table 5:** Ablation study results of TFP-Net components. Results are reported as mean ± std over 5 independent runs.

Model Variant	Taobao User Behavior Dataset			Amazon Product Dataset		
	Accuracy (%)	F1 (%)	Recall (%)	Accuracy (%)	F1 (%)	Recall (%)
Full TFP-Net	87.8±0.3	86.2±0.4	84.7±0.5	88.1±0.2	86.5±0.3	85.0±0.4
w/o TGAT	84.9±0.5	83.1±0.6	81.2±0.7	85.2±0.4	83.3±0.5	81.5±0.6
TGAT-only	82.3±0.7	80.5±0.8	78.9±0.9	82.7±0.6	80.9±0.7	79.2±0.8
w/o TGAT Time Encoding	86.4±0.4	84.6±0.5	82.9±0.6	86.7±0.3	85.0±0.4	83.2±0.5
w/o DeepFM	86.1±0.5	84.5±0.6	82.8±0.7	86.3±0.4	84.7±0.5	83.0±0.6
DeepFM-only	81.5±0.8	79.8±0.9	78.1±1.0	81.9±0.7	80.2±0.8	78.5±0.9
w/o DeepFM FM Layer	87.0±0.4	85.4±0.5	83.8±0.6	87.2±0.3	85.6±0.4	84.1±0.5
w/o ProtoNet	86.9±0.4	85.3±0.5	83.6±0.6	86.7±0.3	85.1±0.4	83.4±0.5
w/o ProtoNet Prototype Gen	87.2±0.4	85.6±0.5	84.0±0.6	87.4±0.3	85.8±0.4	84.3±0.5
ProtoNet-focused Cold-start	83.5±0.6	81.8±0.7	80.2±0.8	82.9±0.7	81.2±0.8	79.6±0.9

## Discussion

The TFP-Net model achieves superior performance on the Taobao and Amazon e-commerce datasets, with outstanding performance in customer behavior analysis, personalized product recommendation and cold-start mitigation, and also exhibits better computational efficiency than baseline models. This verifies the effectiveness of the integrated framework combining temporal graph modeling, deep feature interaction and few-shot prototype learning for CRM system optimization. However, this study still has certain limitations in research design, experimental verification and practical application, which are summarized as follows.

First, the experimental validation is only limited to e-commerce domain datasets, and the unique data characteristics and business logic of e-commerce make the results difficult to be directly generalized to non-e-commerce CRM scenarios such as finance and healthcare, where the model faces prominent cross-industry domain adaptation challenges due to inconsistent data distribution and divergent core task demands. Second, the model’s computational efficiency and scalability are only tested on small-to-medium-sized datasets, with no verification on industrial-level ultra-large-scale CRM datasets; although TFP-Net shows high efficiency in current experiments, it may face practical challenges such as increased training latency and higher memory pressure when processing massive customer behavior data. Third, the cold-start evaluation is confined to controlled 1-shot, 5-shot and 10-shot few-shot settings, lacking exploration of real-world new-user onboarding cold-start scenarios such as zero-sample users with only basic demographic information, leading to limited performance in actual extreme cold-start scenes. Fourth, the model has potential overfitting risks, being prone to overfitting to time-sensitive noise in e-commerce temporal behavior data and small-sample few-shot data in cold-start scenarios, which may affect its generalization ability on unseen data. Fifth, the model only takes structured customer-product interaction data and basic attribute data as input, lacking the integration of multi-modal information such as customer textual reviews and product image features, which limits the capture of comprehensive and fine-grained customer preference features. Sixth, the interpretability analysis of the model (based on attention mechanisms and prototype learning) is superficial: although attention weight distribution and prototype clustering are visualized, there is no in-depth business interpretation of the results, and the practical rationality of the learned interpretability features lacks validation and feedback from e-commerce/CRM domain experts, making the interpretability claims less rigorous.

In view of the above limitations, future research will focus on optimizing TFP-Net from multiple aspects to enhance its practical value and generalization ability in real-world CRM applications. We will collect cross-industry CRM datasets from finance, healthcare and other fields for model verification, and design a lightweight domain-adaptive module based on TFP-Net’s modular framework to learn domain-invariant features, thus solving cross-industry domain adaptation challenges. We will also test the model on ultra-large-scale CRM datasets, and adopt lightweight optimization strategies such as parameter pruning and knowledge distillation combined with distributed training frameworks to ensure its efficient operation in massive data scenarios. For real-world new-user onboarding and extreme cold-start scenarios, we will integrate multi-source external auxiliary information, and explore the combination of prototype learning with generative models and meta-learning to alleviate data scarcity and enhance the model’s fast adaptation ability to new users. To mitigate overfitting risks, we will introduce regularization techniques and data augmentation methods for temporal behavior and cold-start few-shot data to improve the model’s generalization ability. In addition, we will integrate multi-modal learning techniques to extract features from textual reviews, product images and other data, and design a cross-modal feature fusion mechanism to enrich the model’s input and capture more comprehensive customer preferences. We will also conduct in-depth business interpretability analysis of the model’s attention weight distribution and prototype clustering results, and collaborate with e-commerce/CRM domain experts to validate and optimize the interpretability features, ensuring that the learned attention and prototype patterns are consistent with real business logic and practical CRM decision-making needs. Finally, we will further optimize the core modules of TFP-Net, and explore the combination of the model with reinforcement learning and self-supervised learning to enhance its dynamic decision-making ability for customer behavior and feature learning ability with unlabeled data, continuously improving the overall performance and practical adaptability of the model.
